# Engineering by Cuts: How Kirigami Principle Enables Unique Mechanical Properties and Functionalities

**DOI:** 10.1002/advs.202204733

**Published:** 2022-10-30

**Authors:** Jiayue Tao, Hesameddin Khosravi, Vishrut Deshpande, Suyi Li

**Affiliations:** ^1^ Department of Mechanical Engineering Clemson University 224 Fluor Daniel Building, 216 South Palmetto Boulevard Clemson SC 29631 USA; ^2^ Department of Mechanical Engineering Virginia Tech 153 Durham Hall, 1145 Perry Street Blacksburg VA 24060 USA

**Keywords:** buckling, flexible electronics, kirigami, metamaterials, morphing, soft robots, super stretchability

## Abstract

Kirigami, the ancient art of paper cutting, has evolved into a design and fabrication framework to engineer multi‐functional materials and structures at vastly different scales. By slit cutting with carefully designed geometries, desirable mechanical behaviors—such as accurate shape morphing, tunable auxetics, super‐stretchability, buckling, and multistability—can be imparted to otherwise inflexible sheet materials. In addition, the kirigami sheet provides a versatile platform for embedding different electronic and responsive components, opening up avenues for building the next generations of metamaterials, sensors, and soft robotics. These promising potentials of kirigami‐based engineering have inspired vigorous research activities over the past few years, generating many academic publications. Therefore, this review aims to provide insights into the recent advance in this vibrant field. In particular, this paper offers the first comprehensive survey of unique mechanical properties induced by kirigami cutting, their underlying physical principles, and their corresponding applications. The synergies between design methodologies, mechanics modeling, advanced fabrication, and material science will continue to mature this promising discipline.

## Graphical abstract

Kirigami has evolved into a design and fabrication framework for engineering, opening new avenues for building metamaterials, sensors, and soft robotics. This review aims to provide insights into the recent advance in this vibrant field by offering a comprehensive survey of the unique mechanical properties induced by cutting, their underlying physical principles, and their corresponding applications.

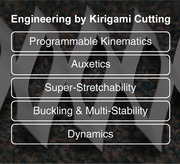

## 1 Introduction

Kirigami—“cut‐paper” in Japanese—is an ancient art of creating beautiful decorations by simply cutting and manipulating a thin piece of paper. The seemingly infinite possibilities of developing 2D or 3D geometries by the kirigami principle have inspired countless implementations in our modern life, from children's pop‐up books to art and architecture. Meanwhile, kirigami received a growing interest from the science and engineering communities, who are transforming this humble artistic activity into a framework for architecting, fabricating, and functionalizing a wide variety of engineered systems like flexible electronics, metamaterials, morphing structures, and soft robotics. The rapidly increasing number of kirigami‐related academic publications over the recent years is a testimony to this exciting development.

**Figure 1:**
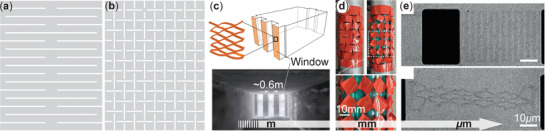
The two archetypal designs of Kirigami and their implementations at different scales. a,b) The design of parallel‐cut and cross‐cut kirigami patterns, respectively. c) A meter‐scale kirigami window shade for commercial buildings. Adapted with permission.^[^ ^13^ ^]^ Copyright 2017, Wiley‐VCH. d) A millimeter‐scale robotic kirigami skin made out of plastic sheets. Reproduced under the terms of the Creative Commons CC‐BY license.^[^ ^14^ ^]^ Copyright 2021, The Authors. Published by Frontiers Media, S.A. e) A micrometer‐scale kirigami spring based on graphene. Adapted with permission.^[^ ^4^ ^]^ Copyright 2015, Springer Nature.

In our traditional perception, kirigami is frequently related to origami (aka “fold‐paper”) due to their apparent similarities. Indeed, one could consider kirigami a variation of origami by allowing cutting in addition to folding. However, the introduction of cuts makes the working principle underpinning kirigami fundamentally different from origami. For example, a periodic and tessellated origami folding pattern turns paper into a kinematically over‐constrained system, reducing the overall degree of freedom. Furthermore, if their facets are stiff, origami could possess only one kinematic degree of freedom, commonly referred to as “rigid folding.” Miura‐ori, which is the foundation of many deployable structures and metamaterials,^[^ ^1^, ^2^, ^3^ ^]^ is a classic example of 1DOF rigid‐foldable origami. On the contrary, kirigami cutting introduces the opposite effect via releasing the continuous constraint in the constituent sheet material and significantly increasing the kinematics degree of freedom. As a result, the kirigami principle is powerful for imparting compliance to inextensible sheet materials, while origami typically creates load‐bearing and space‐filling 3D topologies. Therefore, the application appeal of kirigami‐inspired engineering systems can be quite different from the origami ones. Another unique characteristic of kirigami is its scalability in fabrication and working principle. One can cut and stretch a micrometer‐scale kirigami spring made out of graphene, and it follows a similar working principle as a centimeter‐scale kirigami cutout of paper.^[^ ^4^ ^]^ Such scalability opens up broad appeal across many different disciplines (**Figure** **1**c–e).

**Figure 2:**
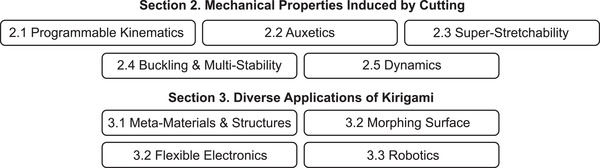
An overview of different topics covered in this paper, including the different cutting‐induced mechanical properties in Section 2 and the diverse applications in Section 3.

While plenty of literature reviews on origami‐inspired engineering systems are available,^[^ ^5^, ^6^, ^7^, ^8^, ^9^, ^10^, ^11^ ^]^ very few literature reviews focus solely on kirigami‐based studies despite the unique working principles mentioned above.^[^ ^12^ ^]^ In particular, there is a critical need for a survey of different mechanical properties created by kirigami cutting and their broad application appeals. This paper aims to fill this gap and provide an extensive progress review and future perspective on this vibrant field. We will first review the unique mechanical properties induced by kirigami cutting and their underlying mechanics, including programmable motion and kinematics, auxetics, super stretchability, buckling, multi‐stability, and dynamics. Then, we will survey the wide use of kirigami‐inspired concepts in metamaterials, multi‐functional structures, electronics, and soft robotics (**Figure** **2**). We will end with a discussion on the current challenges and future outlooks.

## 2 Unique Mechanical Properties Induced by Kirigami Cutting

Among the wide variety of kirigami cutting patterns in the published literature, two strikingly simple designs stand out as archetypal templates. We refer to them as “parallel‐cuts” and “cross‐cuts” hereafter (Figure 1a,b) and use them to explain the physical principles underpinning the cutting‐induced mechanical properties.

### 2.1 Programmable Deformation Kinematics

Without cutting or tearing, manipulation and deformation of thin sheets must satisfy at least *C* ^0^ continuous condition, which imposes strong constraints on the admissible final shapes. Here, *C* ^0^ continuity means any curves on the deformed sheet are continuous without any breaks, meaning there should be no gaps or fractures occurring while deforming the thin sheets. Kirigami cutting essentially releases such constraint by intentionally breaking the *C* ^0^‐continuity. More importantly, a carefully designed cutting pattern can divide a thin sheet into different functional regions—some exhibit limited deformations, while others deform significantly, effectively creating a compliant linkage mechanism. The corresponding linkage's 2D or 3D motion and kinematic characteristics are programmable by simply tailoring the underlying cutting pattern. Therefore, kirigami principles create pathways for generating desired movements for tasks like shape imitation,^[^ ^15^, ^16^ ^]^ morphing structures,^[^ ^17^ ^]^ adaptable electronics,^[^ ^18^ ^]^ and robotic manipulations.^[^ ^19^ ^]^

There are two types of studies regarding kirigami's movement and deformation kinematics. One is global, aiming to achieve global and prescribed 2D or 3D shape change from flat sheets. Such a global shape change is also referred to as morphing. The other is local, aiming to achieve local and controllable movement at specific sites within the kirigami sheet. In this subsection, we review the relevant studies respectively.

#### 2.1.1 Global Shape Change

**Figure 3:**
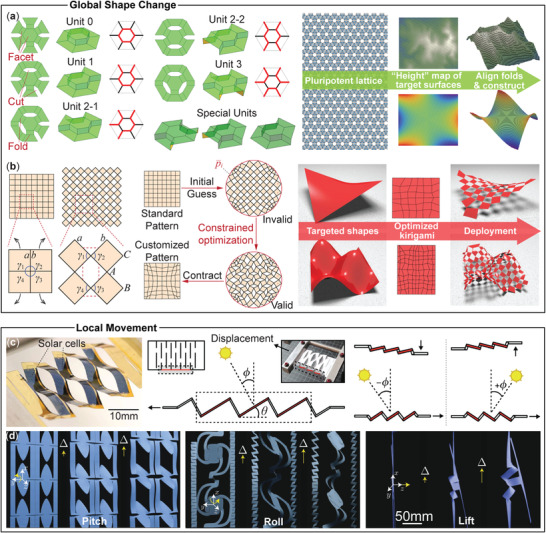
Programming deformation kinematics using the Kirigami principle. a) Morphing with side‐connected, out‐of‐plane rotating kirigami facets. Left: Different building blocks (or units) with steps. Right: By assembling these units into a “pluripotent lattice” and projecting the height of targeted surfaces, one can create complex geometries. Note that this example involves origami folding, but cutting is central for rich deformation kinematics. Adapted with permission.^[^ ^28^ ^]^ Copyright 2015, National Academy of Science. b) Global deformation with corner‐connected, in‐plane rotating kirigami facets. Left: Elementary unit showing the in‐plane rotating facets. Middle: Flowchart of the constrained optimization method. Right: Examples of shape morphing via stretch deployment. Adapted with permission.^[^ ^30^ ^]^ Copyright 2019, The Authors. Springer Nature. c) Local tracking by parallel‐cut kirigami. Lifting, lowering, and stretching the kirigami sheet's free end allows one to orient the embedded solar cell (θ) on demand according to the incoming sunlight angle (ϕ). Reproduced under the terms of the Creative Commons CC‐BY license.^[^ ^18^ ^]^ Copyright 2015, The Authors. Published bySpringer Nature. d) Complex kirigami sheet showing pitch, roll, and lift motion at local sites. Adapted with permission.^[^ ^31^ ^]^ Copyright 2017, Royal Society of Chemistry.

Many published studies have demonstrated the global shape change (morphing) of flat kirigami sheets into prescribed 2D/3D surfaces.^[^ ^8^, ^20^, ^21^, ^22^, ^23^, ^24^ ^]^ The achievable shapes among these studies are complex and vastly diverse. Nevertheless, almost all available morphing methods have leveraged a simple and effective idea at the root level: rotation of polygon facets. By careful cutting, one can divide a continuous sheet into a tessellation of polygon facets connected via their sides or corners, and these connections can be sparse (**Figure** **3**a,b). Such setup provides sufficient kinematic freedoms for these facets to rotate in‐plane and out‐of‐plane and create complex shapes, but the facets themselves deform little in this process. Customizing the cutting pattern changes the geometry of these polygon facets and their kinematic connections, thus customizing the final shape.

If facets connect to their adjacent neighbors via their sides, they can rotate out‐of‐plane around these sides. This is similar to origami folding but with a much larger kinematic freedom. For example, traditional origami sheets show zero‐Gaussian curvature everywhere,^[^ ^25^ ^]^ but with kirigami cutting and folding, 3D shapes with non‐zero Gaussian curvatures can emerge.^[^ ^26^, ^27^ ^]^ By strategically distributing these cuts and folds, one can create building blocks (or units) with “steps” and assemble them into 3D topologies (Figure 3a). Therefore, one can discretize the topography of targeted 3D surfaces into small elements at different heights, and inverse design the corresponding cutting and folding pattern.^[^ ^28^ ^]^

If the polygon facets connect to their adjacent neighbors via their corners, they can rotate in‐plane and out‐of‐plane, exhibiting even richer movements than the side‐connected kirigami sheets mentioned above. As a result, the kirigami sheets can deform in 2D (e.g., from a square to a circular shape) or 3D (e.g., from a flat to a saddle surface) (Figure 3b). It is worth noting that in these corner‐connected kirigami systems, the hinge material connecting adjacent facets typically experiences significant deformation, which can constrain the acceptable morphing deformation range. One can address this problem by introducing “supplemental folds” at these hinges.^[^ ^14^, ^29^ ^]^

Such vast freedom for programming global deformation kinematics by tailoring the cutting pattern necessitates comprehensive and versatile design methodologies, which remain an open research topic. For example, Gary et al. attempted to establish a unifying design optimization framework by evolving from the cross‐cut kirigami (Figure 3b).^[^ ^30^ ^]^ They started from an initial, uniform cross‐cut and then applied constrained optimization on the individual facets toward the targeted final shape. Figure 3b (bottom left) illustrates the elementary unit in the quadrilateral tessellation, showing the four internal angles (γ_*i*_, *i* = 1…4) and the adjacent sides (*a*, *b*). At the initial state, two constraints apply: The summation of all the four internal angles should be 2π, and every pair of edges should have the same length:

(1) $$\begin{array}{cc} \gamma_{1}+\gamma_{2}+\gamma_{3}+\gamma_{4} & =2\pi ,\\ a^{2}-b^{2} & =0 \end{array}$$

After deployment, shape matching constraints are applied at the nodes along the kirigami's periphery, ideally matching the outline of the targeted final shape. Denote the positions of periphery nodal points as *p*_*i*_, and the corresponding target location as $\bar{p_{i}}$, and this constraint becomes

(2) $$\left|\right|p_{i}-\bar{p_{i}}{\left|\right|}^{2}=0$$

In addition, a non‐overlapping constraint during deployment should be applied to the adjacent quadrilateral facets in that

(3) $$\langle \left(B-A\right)\times \left(C-A\right),\hat{n}\rangle \geq 0$$

where *A*, *B*, and *C* are three nodes on adjacent faces, and (*B*, *A*, *C*) form a positive angle between the facets. $\hat{n}$ is the outward unit normal vector (0, 0, 1). Building upon the constraints above, an objective function that facilitates a smooth transition between adjacent quad unit cells can be formulated using: the total number of adjacent pairs of unit cells (*M*), their corresponding pairs of internal sector angles (α_*ij*_, β_*ij*_), and pairs of edge lengths (*a*_*ik*_, *b*_*ik*_) so that

(4) $$\mathrm{Min}\;Z=\frac{1}{M}\sum_{i=1}^{M}\left(\sum_{j=1}^{4}\left(\alpha_{\mathrm{ij}}-\beta_{\mathrm{ij}}\right)+\sum_{k=1}^{4}\left(a_{\mathrm{ik}}-b_{\mathrm{ik}}\right)\right)$$

Minimizing the objective function (*Z*) creates a non‐periodic kirigami cutting pattern that can deform to approximate any sets of 2D/3D surfaces (Figure 3b).

#### 2.1.2 Local Movement

Besides the global shape change, kirigami sheets can also provide controllable motions at designated local sites by exploiting the linkage‐like kinematics from the cutting pattern. In this case, the kirigami sheets typically have long and slender “ligaments” with carefully designed shapes and orientations, and they can deform like a linkage to re‐orient functional sites for different purposes. For example, with mechanical inputs like a simple stretch and lifting at the free end, a parallel‐cut kirigami can orient its facets according to the incoming sunlight angle, achieving dynamic solar tracking and maximized power output (Figure 3c).^[^ ^18^ ^]^ In a different study, Dias et al. achieved more versatile local movements—including roll, pitch, yaw, and lift—by a more elaborate kirigami cutting design (Figure 3d). In particular, this study shows that the shape of the crack tip, either convex or concave, plays a vital role in out‐of‐plane facet rotation.^[^ ^31^ ^]^ Similar principles apply to other electromagnetic systems, as discussed in Section 3 later.

### 2.2 Auxetics

Auxetic behavior (aka negative Poisson's ratio) refers to the unique response that a structure or material can expand, rather than shrink, in the direction perpendicular to an external extension load. Auxetic materials have found numerous applications in electronics,^[^ ^32^ ^]^ metamaterials,^[^ ^33^, ^34^, ^35^ ^]^ robotics,^[^ ^36^, ^37^, ^38^ ^]^ architecture,^[^ ^39^ ^]^ and biomedicine.^[^ ^40^ ^]^ There are two different mechanisms for the kirigami sheet to generate auxetic properties. One is via facet rotations and is directly related to the programmable kinematics reviewed in the previous sub‐section (indeed, auxetics fundamentally is a kinematic property). The other mechanism to achieve auxetics is that kirigami cutting and subsequent folding can develop flat sheets into re‐entrant and open‐celled honeycomb structures. In this subsection, we review and compare the corresponding working principles.

#### 2.2.1 Facet Rotation

**Figure 4:**
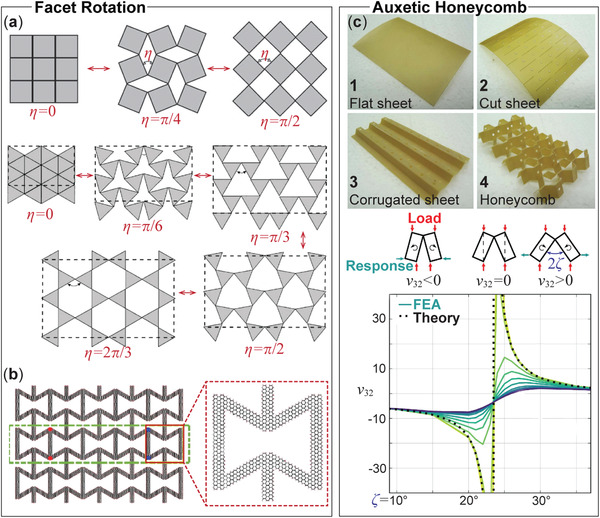
Using the kirigami principle to achieve auxetic properties. a) The rotation of kirigami facets with square or triangular shapes can expand the surface area under tension, giving negative Poisson's ratio in‐plane. Adapted with permission.^[^ ^41^ ^]^ Copyright 2006, Springer Nature. b) Monolayer graphene cut into a re‐entrant honeycomb shape. Reproduced under the terms of the Creative Commons CC‐BY license.^[^ ^43^ ^]^ Copyright 2016, The Authors. Published by Springer Nature. c) An auxetic, open‐celled kirigami honeycomb core. Pictures on the top show its fabrication process based on cutting and then folding. The side views in the middle illustrate its unit cell shapes at different folding configurations (described by the angle ζ), showing the deformation mechanisms of negative, zero, and positive Poisson's ratio out‐of‐plane. The figure at the bottom summarizes the open‐celled kirigami honeycomb's Poisson's ratio at different folding configurations. Reproduced under the terms of the Creative Commons CC‐BY license.^[^ ^17^ ^]^ Copyright 2016, The Authors. Published by Springer Nature.

The cross‐cut kirigami could produce 2D surfaces with a negative Poisson ratio. Here, the individual facets may vary in shape and size, but they can exhibit complex and intricate rotations that make the surface grow (or shrink) in response to an in‐plane stretch (or compression) (**Figure** **4**a). Two examples were presented by Grima et al.,^[^ ^41^, ^42^ ^]^ where they designed the auxetic behavior by assuming rigid facets in one case and compliant facets in the other. The Poisson's ratio is independent of the facets' size but dependent on their rotation angles, creating Poisson's ratio fluctuating between 0 and −1.

#### 2.2.2 Auxetic Honeycomb by Kirigami

Many variations of cellular honeycomb structure have shown auxetic behaviors, such as the re‐entrant honeycomb,^[^ ^43^ ^]^ open‐honeycomb,^[^ ^17^, ^44^ ^]^ chiral‐honeycomb,^[^ ^45^ ^]^ SILICOMB,^[^ ^46^, ^47^ ^]^ and AUXHEX.^[^ ^48^ ^]^ Kirigami cutting and folding provide a simple way to create these honeycomb cores from flat constitutive sheets. For example, the re‐entrant honeycomb is an inverted version of the traditional hexagonal honeycomb, where the angle between two side walls is acute. By inspecting the deformation characteristics for a unit cell in this re‐entrant honeycomb, one observes that the sidewall inverted inside exerts an inward force in response to in‐plane compression. As a result, when the re‐entrant honeycomb is compressed in one in‐plane direction, it shrinks in other in‐plane directions. Such a re‐entrant honeycomb concept is easily scalable for different constituent materials. For example, even a highly stiff material such as graphene could become an auxetic surface by introducing a re‐entrant kirigami cutting pattern (Figure 4b).^[^ ^43^ ^]^

The open honeycomb is a honeycomb variation made by cutting and folding flat sheets (Figure 4c).^[^ ^17^ ^]^ This concept has been applied to different constitutive sheet materials for different purposes, like shape memory polymers for deployable surfaces,^[^ ^44^ ^]^ metal and fiber composites for crushable cruciforms,^[^ ^49^ ^]^ as well as polyether‐ether‐ketone (PEEK) for its ease of manufacturing.^[^ ^46^ ^]^ The open honeycomb configuration is quite versatile. When fully folded and pasted, it converges to the traditional honeycomb core, but it could also unfold to generate rich geometries that exhibit a programmable Poisson's ratio. The open honeycombs are also monoclastic: Its bending curvature in one direction does not affect the other, making them suitable for angular deployment mechanisms.

Another honeycomb variation called SILICOMB was developed by Scarpa et al.^[^ ^50^ ^]^ Its unit cell consists of eight facet linkages with two reflex angles subtended by opposite corners and at least two obtuse angles. The SILICOMB concept could be used to create flat^[^ ^44^, ^46^, ^50^ ^]^ or curved sandwich structures^[^ ^51^ ^]^ depending on the mold geometry used during fabrication. The flat SILICOMB sandwich—made by PEEK for its potential for aerospace applications—showed very low or close to zero Poisson's ratio with negligible synclastic (surfaces that have centers or curvatures on the same side) and anticlastic (surfaces that have centers or curvatures on the opposite side, i.e., saddle shape) behaviors.^[^ ^47^ ^]^

### 2.3 Super‐Stretchability

Super‐stretchability (also known as super‐elasticity) refers to the ability to sustain substantial deformation, sometimes with more than 100% strain, and return to its original shape after removing the external load. Achieving such super‐stretchability typically requires a structure or material system to be folded, bent, and stretched without damage or loss of original functions. Traditionally, super‐stretchability is limited to elastomeric materials like rubber. Many functional materials, like electronics and composites, cannot be stretched significantly despite the clear advantages of doing so. For example, traditional batteries are stiff, and deforming them creates hazardous conditions.^[^ ^52^ ^]^ However, flexible and stretchable batteries could enable many new capabilities in soft robotics^[^ ^53^ ^]^ and wearable electronics devices.^[^ ^54^ ^]^ To this end, kirigami cutting offers a scalable and easy‐to‐fabricate approach to impart stretchability far beyond the constituent sheet materials. There have been many successful implementations on very stiff constitutive materials like graphene,^[^ ^4^, ^55^, ^56^ ^]^ boron nitride (h‐BN) nano‐sheets,^[^ ^57^ ^]^ MoS2 monolayer,^[^ ^58^, ^59^ ^]^ metal,^[^ ^60^ ^]^ and 2D phosphorene.^[^ ^61^ ^]^ As a result, super‐stretchable kirigami materials have shown broad application appeals, such as architectures,^[^ ^62^ ^]^ shape‐morphing systems,^[^ ^63^, ^64^ ^]^ flexible electronics,^[^ ^65^, ^66^, ^67^, ^68^, ^69^ ^]^ sunlight tracking and wearable solar cell,^[^ ^18^, ^70^ ^]^ robotic actuators,^[^ ^14^, ^71^ ^]^ optics,^[^ ^72^ ^]^ stretchable heaters,^[^ ^73^ ^]^ energy harvester,^[^ ^74^, ^75^ ^]^ bioprobe,^[^ ^76^ ^]^ artificial skin,^[^ ^77^ ^]^ telecommunication devices,^[^ ^78^ ^]^ and adaptive transparent surfaces.^[^ ^79^ ^]^ We will detail some of these applications in Section 3. Here, we focus on the mechanical principles underpinning kirigami's super‐stretchability.

Kirigami sheets have two different mechanisms for creating super‐stretchability. By the first mechanism, the cutting patterns allow the constituent sheets' deformation to concentrate at pre‐determined local sites (usually near the tip of the cuts) while keeping the rest of the sheet relatively undeformed. Stretching deformation, in this case, is usually in‐plane. On the other hand, the second mechanism of super‐stretchability involves out‐of‐plane deformation in that the kirigami cutting pattern can convert a global stretching into local bending.

#### 2.3.1 In‐Plane Mechanism

**Figure 5:**
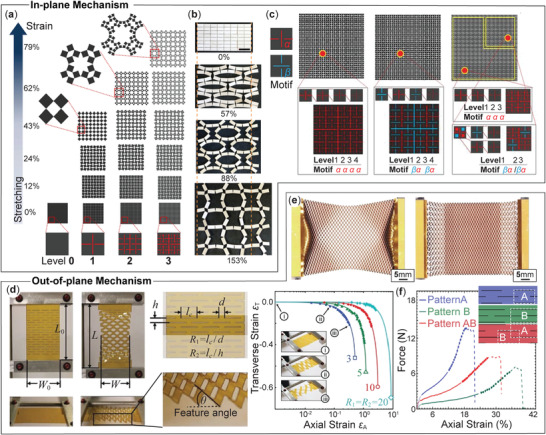
Using the kirigami principle to impart super‐stretchability to stiff sheet materials by either in‐plane (a–c) or out‐of‐plane mechanisms (d–f). a) Facet rotations in cross‐cut kirigami offer a versatile route to stretchability, especially when the cutting pattern is hierarchical. Adapted with permission.^[^ ^80^ ^]^ Copyright 2014, National Academy of Science. b) Adjusting the facets' shape, such as the aspect ratio, can increase the stretchability significantly. Reproduced under the terms of the Creative Commons CC‐BY license.^[^ ^82^ ^]^ Copyright 2017, The Authors. Published by Elsevier. c) By strategically combining different cut motifs at different hierarchical levels, one can prescribe the stretchability with large freedom. Adapted with permission.^[^ ^80^ ^]^ Copyright 2014, National Academy of Science. d) Out‐of‐plane rotation and bending of kirigami facets offer another route to stretchability, which can also be prescribed by tailoring the cut pattern designs. Reproduced under the terms of the Creative Commons CC‐BY license.^[^ ^18^ ^]^ Copyright 2015, The Authors. Published by Springer Nature. e) A non‐uniform cut pattern eliminates the constraints from fixed boundaries. Reproduced under the terms of the Creative Commons CC‐BY license.^[^ ^84^ ^]^ Copyright 2021, The Authors. Published by MDPI. f) A hybrid kirigami pattern—combining a cutting design A with strong loading capacity and another design B with high stretchability—gives a balanced performance. One can also observe that these force‐deformation curves are strongly nonlinear. Adapted with permission.^[^ ^85^ ^]^ Copyright 2020, American Chemical Society.

This mechanism is directly related to the linkage‐like kinematics created by kirigami cuts. For example, suppose a sheet material is cut by a tessellation of cross‐shaped cutting motifs and placed under in‐plane stretching. In this case, its joints (or hinges) near the cut tips experience significant and concentrated deformation. However, the facets (or links) are approximately strain‐free and only show rigid‐body rotations (**Figure** **5**a).^[^ ^80^ ^]^ Therefore, if the hinges are sufficiently flexible and the functional components (e.g., electronics) are on the facets, the kirigami sheet can show super‐stretchability with multiple functionalities.^[^ ^81^ ^]^

One can further enrich the stretching performance space by introducing hierarchy into the kirigami cut pattern: dividing a facet in the kirigami sheet into smaller facets via more minor cuts, creating linkage kinematics at multiple levels. For example, fractal cross‐cuts enable a kirigami sheet to expand to more than 800% of its original area with only very minor stretching of the underlying material (Figure 5a). Moreover, adjusting the cuts' lengths (or facets' aspect ratio) is another powerful method to program stretchability. For example, by increasing facets' aspect ratio from 1 : 1 (aka square shape) to 2 : 1, one can dramatically increase the theoretically maximum stretch from 41% to 124% for a 1‐level cut pattern without hierarchy and from about 62% to 156% for a 2‐level hierarchical cut pattern (Figure 5b).^[^ ^82^ ^]^ Surprisingly, permuting the cutting pattern arrangement can also change the in‐plane stretchability. For example, Cho et al. presented a hierarchical kirigami sheet design consisting of two motifs, α β, with the same cut length and aspect ratio but rotated by 90 degrees (Figure 5c). Denote ϵ_*N*_(*x*_1_ *x*_2_…*x*_*N*_) as the maximum stretch for an *N*‐level hierarchical cut pattern with a motif arrangement of *x*_1_ *x*_2_…*x*_*N*_, where *x*_*i*_ = α or β. One found that $\varepsilon_{4}\left(\alpha \alpha \alpha \alpha \right)=\varepsilon_{4}\left(\beta \beta \beta \beta \right)=108\%$, but for the alternating motif arrangement, $\varepsilon_{4}\left(\beta \alpha \beta \alpha \right)=130\%$. Therefore, the versatility of tuning and combining different design features in kirigami cut pattern (aka hierarchy, aspect ratio, motif arrangement) can dramatically expand the design space for a wide variety of applications.^[^ ^83^ ^]^

#### 2.3.2 Out‐of‐Plane Mechanism

Kirigami cuts also create the kinematics freedom for the facets to rotate or bend out‐of‐plane, offering another mechanism for super‐stretchability. For example, the square facets in the cross‐cut kirigami sheet can also rotate out of the plane under a sufficiently large in‐plane stretching;^[^ ^86^ ^]^ and the slender facets in the parallel‐cut kirigami can rotate and bend in 3D^[^ ^8^, ^16^, ^18^ ^]^ (Figure 5d). The latter example can be advantageous because the stretch‐induced deformation is uniformly distributed via facet bending rather than concentrated near the hinges, offering a potentially more resilient mechanical performance.

Consider the simple kirigami structure consisting of a linearly cut pattern in a continuous sheet.^[^ ^18^ ^]^ The out‐of‐plane deformation of the facets creates a “feature angle” θ between the tilted facets and the reference plane (Figure 5d). Some kinematics analysis can reveal the correlation between this feature angle, transverse strain ε_*T*_, and longitudinal strain ε_*A*_ of the kirigami sheet in that:

(5) $$\theta =\cos^{-1}\frac{1}{\varepsilon_{A}+1};\varepsilon_{T}=\frac{R_{1}-1}{R_{1}+1}\left[\cos\;\left(\sin^{-1}\frac{2R_{1}\tan\theta }{R_{1}R_{2}-R_{2}}\right)-1\right]$$

where ε_*A*_ = (*L* − *L*_0_)/*L*_0_, and ε_*T*_ = (*W* − *W*_0_)/*W*_0_. *R*_1_, *R*_2_ are dimensionless parameters that define the cut pattern in that *R*_1_ = *l*_*c*_/*d* and *R*_2_ = *l*_*c*_/*h*. Here, kirigami cut designs involve the cut length (*l*_*c*_) and the spacing between cuts in the transverse (*d*) and longitudinal (*h*) directions. This analytical relationship among θ, ε_*T*_, and ε_*A*_ was verified experimentally based on several kirigami samples with *R*_1_ = *R*_2_ = 3, 5, 10, and 20 (right half of Figure 5d). Larger *R*_1_ and *R*_2_ values give more dense cut patterns and higher ϵ_*A*_ and ϵ_*T*_ before fractures occur at the cut tip.

Like the in‐plane mechanism mentioned above, tailoring the kirigami cut design features can also program the out‐of‐plane super‐stretching mechanism. For example, adding “minor cuts” to the tips of “major cuts” introduces new boundary conditions, reducing the kirigami sheet's stiffness by a factor of 30 and increasing ultimate strain by a factor of 2 (up to 750% strain).^[^ ^87^ ^]^ Another unique design feature is the nonuniform cutting pattern, which is particularly useful to mitigate undesirable boundary effects.^[^ ^88^ ^]^ If the kirigami sheet with a uniform cut pattern is fully fixed at both ends during stretching, its deformation at the boundary is constrained by the rigid fixtures. Taniyama et al. addressed this issue by introducing non‐uniform cut patterns—using progressively more trapezoidal facet shapes near the boundaries and adding separation cuts—to increase the stretchability (Figure 5e).^[^ ^84^ ^]^

Super‐stretchability, ultimately, is constrained by the plastic deformation and fracture failure near the tips of the cuts.^[^ ^89^, ^90^ ^]^ One can fine‐tune the cutting pattern design to mitigate or delay the occurrence of these plastic responses and fractures. For example, one can widen the cuts and use rounded cut corners,^[^ ^91^ ^]^ reduce cut spacing,^[^ ^74^ ^]^ or use hierarchical cuts.^[^ ^33^ ^]^ For example, Dijvejin et al. proposed a heterogenous pattern combining two homogenous patterns, A and B, with different geometric designs and found that it can provide a balance between stretching ability and ultimate load‐capacity (Figure 5f).^[^ ^85^ ^]^

### 2.4 Buckling and Multi‐Stability

Large deformations in the kirigami sheets, especially the facets' rotation, create geometry‐induced nonlinearity. For example, the stiffness of super‐stretchable kirigami is rarely linear, as evidenced by the force‐displacement curves in Figure 5f. This section discusses two closely‐related nonlinear properties emerging from stretching the kirigami sheet: buckling and multi‐stability.

#### 2.4.1 Buckling

Buckling typically occurs when a slender structure is under longitudinal compression. As the compression reaches a critical level, elastic instability occurs at the original equilibrium, forcing the structure to deform laterally to new stable equilibria. Surprisingly, kirigami sheets can exhibit buckling under global tension. This unusual phenomenon originates from the cut pattern's underpinning kinematics that can “invert” the external in‐plane stretch into localized compression on the facets. As a result, when the in‐plane global stretch reaches a critical level, the kirigami sheets buckle—at the hinge or the facets, depending on the cutting pattern design—and exhibit out‐of‐plane deformations.

**Figure 6:**
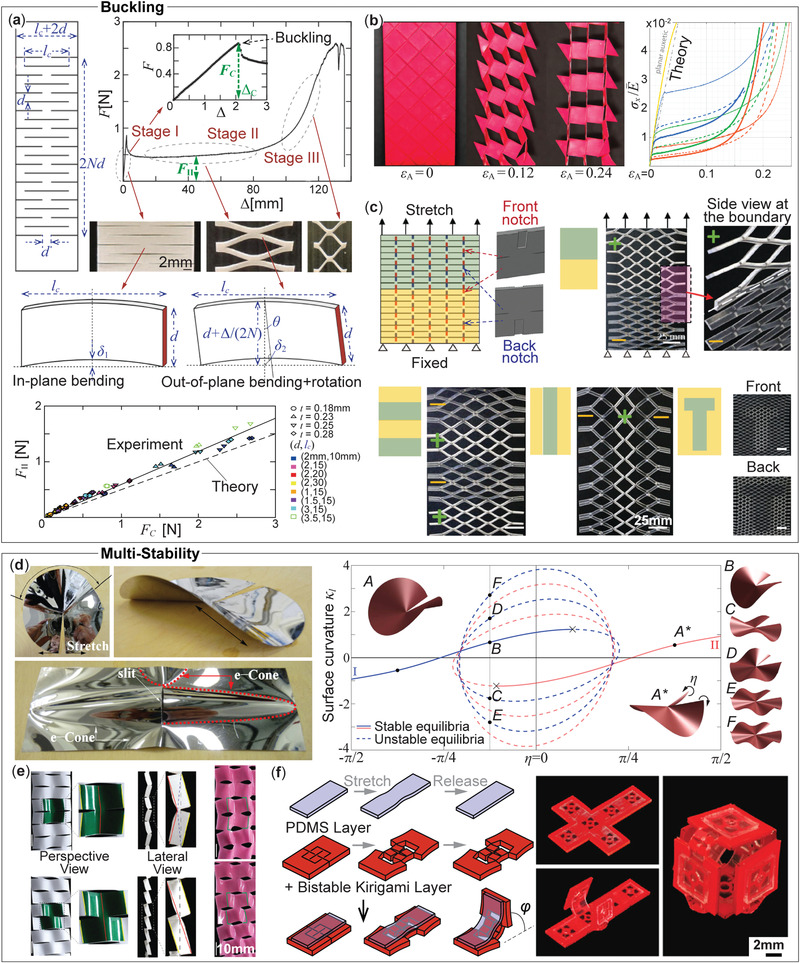
Buckling and multi‐stability in kirigami. a) A typical stretching force‐deformation curve of a parallel‐cut kirigami shows three distinct stages, each corresponding to a unique deformation mechanism. Buckling occurs at the transition from stage I to II. Reproduced under the terms of the Creative Commons CC‐BY license.^[^ ^92^ ^]^ Copyright 2016, The Authors. Published by Springer Nature. The bottom figure shows the corresponding correlation buckling force *F*_*C*_ is directly related to cutting pattern design. Reproduced under the terms of the Creative Commons CC‐BY license.^[^ ^93^ ^]^ Copyright 2019, The Authors. Published by American Physical Society. b) Cross‐cut kirigami can show similar three‐staged nonlinear responses, and out‐of‐plane buckling. Adapted with permission.^[^ ^86^ ^]^ Copyright 2017, American Physical Society. c) Adding notches to the front or back side of parallel‐cut kirigami (aka “Kiri‐kirigami”) is an easy method to control the out‐of‐plane buckling direction. Adapted with permission.^[^ ^13^ ^]^ Copyright 2016, Wiley‐VCH. d) The external shape of an e‐cone near the kirigami cut tip. Adapted with permission.^[^ ^23^ ^]^ Copyright 2016, American Physical Society. The figure on the right shows its equilibrium graph with many static equilibria, two of which are stable. Adapted with permission.^[^ ^94^ ^]^ Copyright 2021, Elsevier. e) By exploiting the bi‐stability of the e‐cone, one can achieve reversible property programming by switching between different stable equilibria. Adapted with permission. Copyright 2018, American Physical Society. f) Cross‐cut kirigami can also be bi‐stable in plane.^[^ ^96^ ^]^ By combining the bi‐stable kirigami layer with a mono‐stable PDMS layer, one can make bistable hinges that can be assembled for complex shape changes. Reproduced under the terms of the Creative Commons CC‐BY license.^[^ ^96^ ^]^ Copyright 2020, The Authors. Published by Elsevier.

Consider a parallel‐cut kirigami sheet under in‐plane tension, its typical force‐displacement curves exhibit three consecutive stages (**Figure** **6**a).^[^ ^92^ ^]^ In the first stage I, the reaction force rises quickly as all facet deformations are in‐plane. Buckling occurs when the in‐plane stretch reaches a critical level, where the slender facets start rotating and bending out‐of‐plane. At this transition point, the force‐displacement curve shows a sharp change in slope or even discontinuity.^[^ ^93^ ^]^ The kirigami sheet then enters the second stage II with a much more gentle rise of reaction force, showing a locally deformed rhomboid pattern. In fact, this second stage corresponds to the super‐stretchability reviewed in the previous subsection. When the kirigami sheet is stretched further into the third stage III, it starts to develop plastic deformation, and its facets are stretched directly. As a result, the reaction force rises rapidly again with strongly nonlinear characteristics until failure. It is worth noting that besides the simple parallel cuts, other cutting patterns like the orthogonal cross‐cuts also induce buckling under stretching, and they exhibit similar three‐staged nonlinear responses (Figure 6b).^[^ ^86^ ^]^ In this case, the buckling deformation concentrates near the hinges. In the reverse case, the parallel‐cut design could also be used to relieve the stresses induced in elastic materials.^[^ ^97^ ^]^

Isobe et al. carefully examined the buckling between the first stiff and the second super‐stretchable stage and formulated a simple scaling theory.^[^ ^92^ ^]^ This theory suggests that the facets' in‐plane bending energy competes with the out‐of‐plane bending energy, and buckling occurs when the two energies become equal. Consider the kirigami sheet as a serial assembly of 2*N* segments characterized by the cut length *l*_*c*_ + 2*d* ≈ *l*_*c*_ (i.e., *l*_*c*_ ≫ *d*), cut spacing *d*, and sheet thickness *t* (Figure 6a_1_). Denote Δ as the overall in‐plane stretch. Based on the small deflection theory of thin plate, the in‐plane bending energy corresponding to the first stiff stage I is

(6) $$U_{I}\left(\Delta \right)≃2\mathrm{NE}d^{3}t\delta_{1}^{2}/l_{c}^{3},\;\mathrm{for}\;\delta_{1},d\ll l_{c}$$

where δ_1_ = Δ/(2*N*). The out‐of‐plane energy, corresponding to the super‐stretchable stage II, is

(7) $$U_{\mathrm{II}}\left(\Delta \right)≃2\mathrm{NE}t^{3}d\delta_{2}^{2}/l_{c}^{3},\;\mathrm{for}\;\delta_{2},d\ll l_{c}$$

where δ_2_ is the out‐of‐plane deflection in that $\delta_{2}^{2}={\left(\Delta /2N+d\right)}^{2}-d^{2}$.

Buckling occurs when *U*_I_(Δ) = *U*_II_(Δ), which yields the critical in‐plane stretch Δ_*c*_:

(8) $$\Delta_{c}=2N\delta_{c},\;\mathrm{where}\;\delta_{c}=\frac{2t^{2}d}{d^{2}-t^{2}}$$

Experiment results from kirigami samples with different cutting pattern designs validated this simple scaling theory, whereas a slight discrepancy occurs when *l*_*c*_ ≪ 5*d* (Figure 6a_2_). This inconsistency is due to small deflection assumptions in the bending energy formulation in Equations (6) and (7). In other words, this simple scaling theory gives accurate prediction when *l*_*c*_ ≫ *d*.^[^ ^92^, ^98^ ^]^

In the classical column buckling problem, the column structure can randomly deform toward any lateral directions when the compression load reaches the critical level. Such randomness also occurs for kirigami buckling. In the parallel‐cut kirigami example, one can observe its post‐buckled facets rotating in either out‐of‐plane directions randomly (clockwise or counter‐clockwise viewed from the side), depending on the mechanical imperfections.^[^ ^99^ ^]^ However, we can prescribe the post‐buckling direction by introducing notches next to the cuts.^[^ ^13^ ^]^ This idea is referred to as the “Kiri‐kirigami.” Figure 6c shows the kiri‐kirigami design with patterned notches on both sides, which generates a deterministic tilting orientation upon uniaxial stretches. One can further program its deformation mechanisms and mechanical properties by using these notches to direct the buckling direction within kirigami – either homogeneously or non‐homogeneously. Also, parallel‐cut design modifications could yield enhanced buckling performance that transcends into high stretchability and flexibility.^[^ ^100^ ^]^

#### 2.4.2 Multi‐Stability

In the Kiri‐kirigami study mentioned above, the two admissible out‐of‐plane buckling directions (aka clockwise or counter‐clockwise rotation) indicate the existence of multi‐stability in stretch buckled kirigami sheet. As the name suggests, multistability refers to the ability of a structure or material system to settle itself in more than one configuration without any external aid. Multistability can originate from the strong nonlinearities in constitutive material properties or motion kinematics. An example of the former case is asymmetric carbon fiber reinforced composite polymers (CFRP). A simple, two‐layer CFRP patch with [0°/90°] ply layout exhibits two stable states with inverted curvatures due to the differential internal stress developed during curing. These states could be switched back and forth by a snap‐through process.^[^ ^101^ ^]^ Combining multiple patches in a kirigami‐inspired pattern can create sophisticated shape morphing and property programming.^[^ ^102^ ^]^ However, it is also possible to exploit the deformation kinematics of kirigami to achieve multi‐stability regardless of the constitutive materials.

Let us consider a simple straight cut in a sheet of rectangular paper (Figure 6d). If this paper is stretched in‐plane and perpendicular to the cut, it deforms itself out‐of‐plane near the cut tip, induced by the buckling phenomena discussed in the previous subsection. The stress near the cut forces the paper to develop a peculiar dome‐like cone called “e‐cones.” The e‐cones get their name due to the phenomenon observed when an angular excess is introduced in the slit cut geometry; this could be understood using the disk geometry in Figure 6d, where the stretch in the circumferential direction about a slit cut forces a part of the disk to deform out‐of‐plane. Such an e‐cone can develop toward either side of the kirigami sheet, generating bi‐stability. Sadik et al. conducted an in‐depth analysis of the nonlinear mechanics underpinning such bi‐stable e‐cone system.^[^ ^94^ ^]^ Seffen et al. further used it to create an interesting “k‐cone” folding geometry for simple shape morphing.^[^ ^23^ ^]^ Yang et al. applied such bi‐stability in a parallel‐cut kirigami sheet to achieve reversible mechanical property programming (Figure 6e).^[^ ^95^ ^]^

Cross‐cut kirigami could also exhibit multi‐stability with desirable 3D shapes. A simple unit of cross‐cut kirigami consisting of four facets could be made into a bi‐stable switch. In the initial stable equilibrium, all four facets are in their initial position, connected at their corners via the hinges. These hinges allow the facets to rotate in response to tensile load, reaching the other stable equilibrium (Figure 6f).^[^ ^96^ ^]^ One can further enrich such multi‐stability by bonding the kirigami sheet to an elastic layer. Such cross‐cut kirigami also found application in mechanical computing by creating logic gates using their multi‐stability.^[^ ^103^ ^]^ Besides the parallel and cross‐cuts, one could implement other cut designs to create different kinds of multi‐stability,^[^ ^95^ ^]^ building upon the phenomenon of stress‐induced multi‐stability near cut tips.

### 2.5 Dynamics

Unlike the static mechanical properties discussed in the previous sections, the dynamic response of kirigami sheets is still an open research topic with few published studies. So far, two dynamic properties have been investigated: mechanical wave propagation control and low‐velocity impact absorption. This section briefly discusses the corresponding working principles.

#### 2.5.1 Wave Propagation Manipulation

The studies on wave manipulation in kirigami are inspired by the vibrant studies on mechanical metamaterials, which exploit their micro‐architectures to derive unorthodox behaviors in phononic wave propagation. This is because the phononic wave's behaviors depend on the inertia and elasticity distributions in the propagation media. If one can prescribe these distributions via carefully designing the underlying micro‐architecture, we can control wave dynamics – such as wave speed,^[^ ^104^ ^]^ propagating direction,^[^ ^105^ ^]^ dispersion,^[^ ^106^ ^]^ and localization properties,^[^ ^107^ ^]^ with considerable freedom. For example, metamaterials with periodic micro‐architectures can exhibit bandgaps: a frequency range where phononic wave propagation completely stops due to Braggs scattering^[^ ^108^ ^]^ or local resonance.^[^ ^109^ ^]^

However, fabricating such periodic architectures can be challenging and involve complex manufacturing techniques.^[^ ^110^ ^]^ To this end, kirigami cutting and subsequently stretching (or folding) provides a scalable and straightforward approach to designing and fabricating metamaterials.

**Figure 7:**
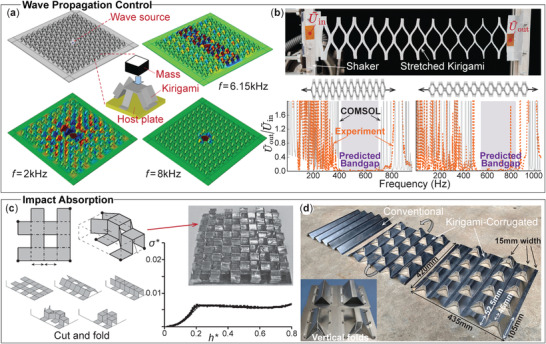
Wave propagation control (a,b) and impact absorption (c,d) in kirigami. a) A meta‐structure consisting of a periodic array of resonant mass attached to a host plate via kirigami fixtures. It can provide a directional flexural wave propagation guide at 6.15 kHz or a total wave propagation stopband at 8 kHz. Reproduced under the terms of the Creative Commons CC‐BY license.^[^ ^111^ ^]^ Copyright 2018, The Authors. Published by Springer Nature. b) Parallel‐cut kirigami can also provide Braggs wave bandgap, and the stop band frequency is easily tunable by controlling the stretch. Adapted with permission^[^ ^113^ ^]^ Copyright 2022, American Physical Society. c,d) Kirigami cut and fold is a simple and versatile method to fabricate lightweight structures for impact absorption, such as the cube core^[^ ^114^ ^]^ and corrugated kirigami.^[^ ^115^, ^116^ ^]^ Notice that the vertical folds in the insert figure can significantly improve the impact protection. Adapted with permissions.^[^ ^114^, ^115^, ^116^ ^]^ 2015, 2020, 2021, Elsevier.

For example, Zhu et al. proposed kirigami‐inspired metamaterial with a no‐perforation design and subwavelength flexural wave manipulation capability (**Figure** **7**a).^[^ ^111^ ^]^ By attaching “resonant kirigami” structures periodically on the top of the host plate, one can construct a metamaterial system without any perforations to avoid degrading the overall mechanical strength. Moreover, such a setup offers an anisotropic effective mass density, stemming from coupling the kirigami cell's local resonance with the host plates' global flexural wave propagations. Ouisse et al. used the kirigami cut and fold principle to construct an auxetic pyramidal lattice core with shunted piezoelectric patches.^[^ ^112^ ^]^ The shunt circuitry with negative capacitance created a very large bandgap in the low‐frequency range. Li et al. designed a family of folded kirigami by replacing the point‐like hinges in the conventional kirigami with 3D folded hinges.^[^ ^49^ ^]^ Such a hinge folding mechanism enables a unique “polarization switch”—the kirigami would expand first and then shrink to smaller than its original size. The polarization switch creates unorthodox properties like peak nominal strain, stiffness singularity, and tunable phononic bandgaps that can shift in response to hinge folding. Finally, Khosravi and Li examined the Braggs bandgap and its mechanical tuning in a stretch‐buckled parallel‐cut kirigami sheet (Figure 7b).^[^ ^113^ ^]^ Their theoretical calculation, numerical simulation, and experiments confirmed the transverse elastic wave propagation bandgaps and their correlation to simple stretching. These studies opened an avenue for using kirigami as a simple and effective approach for creating and adapting periodicity for wave propagation control.

#### 2.5.2 Impact Absorption

The impact absorption performance of the kirigami‐inspired structures relies on their 3D corrugated shapes. As the tesselation of kirigami facets rotates out‐of‐plane via stretching and folding, they create sandwich core‐like structures. When impact force occurs, these kirigami facets deform plastically to absorb the impact energy, protecting the systems underneath.

Since the kirigami facets' shape and orientation directly dictate the impact absorption results, many different kirigami‐enabled core structures have been designed, including eggbox and cube shapes,^[^ ^114^ ^]^ modified corrugation,^[^ ^115^, ^116^ ^]^ cruciform,^[^ ^117^ ^]^ and square dome^[^ ^118^ ^]^ (Figure 7c,d). Among these designs, two interesting features emerge. One is that vertical folds (highlighted in the figure) can significantly increase the absorbed impact energy. The other is that additional cutouts can be intentionally placed on the kirigami facets to reduce the impact peak force.

## 3 Diverse Applications of Kirigami

### 3.1 Meta‐Materials and Multi‐Functional Structures

Metamaterials (and meta‐structures at a larger scale) derive their mechanical properties primarily from their underlying architectures rather than constitutive material properties. They are typically assemblies of smaller‐scale units with purposefully designed geometry. By exploiting the correlations between geometry and mechanical properties, metamaterial and metastructures can achieve unorthodox properties unseen in traditional systems,^[^ ^119^ ^]^ such as extremely high strength‐weight ratio,^[^ ^120^ ^]^ wave propagation manipulation,^[^ ^121^ ^]^ and negative constitutive properties (e.g., negative Poisson's ratio,^[^ ^122^ ^]^ negative stiffness,^[^ ^123^ ^]^ negative thermal expansion coefficient^[^ ^124^ ^]^).

The cutting‐induced mechanical properties discussed in the previous section follow the same geometry‐property correlations. Therefore, kirigami is fundamentally a metamaterial (or metastructure), offering a broad appeal for many mechanical applications at different scales, such as morphing aircrafts,^[^ ^125^, ^126^ ^]^ cruciforms in marine vessels,^[^ ^117^ ^]^ sandwich core structures,^[^ ^47^, ^48^, ^116^, ^118^ ^]^ window shades,^[^ ^13^ ^]^ and inflatable devices.^[^ ^15^ ^]^ Since we have discussed these mechanical functions throughout Section 2, we will not dedicate more space here for brevity.

### 3.2 Active Morphing Surface

**Figure 8:**
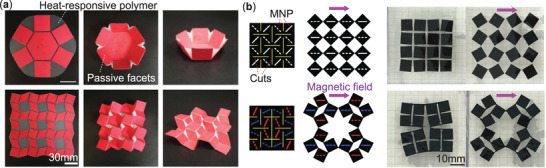
Self‐actuated kirigami morphing surface with (a) embedded heat‐responsive polymers. Adapted with permission.^[^ ^127^ ^]^ Copyright 2018, Wiley‐VCH. And (b) embedded magnetic nanoparticle chains (MNP). Adapted with permission.^[^ ^37^ ^]^ Copyright 2020, American Chemical Society.

Kirigami sheets can be developed into morphing surfaces by leveraging the programmable deformation kinematics reviewed in Section 2.1. Moreover, one can integrate different actuation methods to make such morphing autonomous. To this end, one can rely on external mechanical inputs like a simple stretch,^[^ ^128^ ^]^ yet embedded actuation also has unique advantages. One promising method to achieve self‐actuated morphing is by embedding responsive materials.^[^ ^129^, ^130^, ^131^, ^132^ ^]^ For example, Cui et al. used a heat‐responsive polymer sheet (polystyrene) as the base layer and attached stiff facets to create a self‐morphing kirigami.^[^ ^127^ ^]^ Without the stiff facets, the underlying polystyrene sheet would shrink uniformly above the glass transition temperature (around 150 °C). However, the constraints from the stiff kirigami facets generate local and nonuniform shrinkage, forcing the kirigami sheet to deform into a 3D surface. Based on the facets' arrangement, one can create and combine two different kinds of “gadgets,” each with unique deformation characteristics (**Figure** **8**a).

Another promising concept is magnetic actuation because it is fast, safe, and easy to control.^[^ ^19^, ^133^, ^134^, ^135^ ^]^ Magnetic actuation can be achieved by embedding magneto‐responsive materials, such as magnetorheological elastomers (MRE)^[^ ^136^ ^]^ and Fe‐PDMS, in the host kirigami facets. For example, Jiralerspong et al. demonstrated a magnetic‐driven cross‐cut kirigami with embedded magnetic nanoparticle chains (MNP) (Figure 8b).^[^ ^37^ ^]^ The kirigami facets, in this case, rotate themselves to align the embedded magnetic particle chains to the direction of external magnetic fields, generating 2D or 3D shape transformations.

Other self‐actuation strategies for kirigami involve using shape memory alloy (SMA) actuators,^[^ ^137^, ^138^ ^]^ or soft pneumatic actuators.^[^ ^14^, ^139^, ^140^ ^]^ Integrating the design and self‐actuation methods can provide a simple yet powerful method to program morphing surfaces.

### 3.3 Flexible Electronics

However, it is crucial to point out that kirigami is not limited to mechanical functions. One can make kirigami‐based metamaterials with tunable thermal^[^ ^141^ ^]^ or electro‐magnetic properties.^[^ ^142^ ^]^ More importantly, kirigami could be coupled with different electrical or biochemical components to create next‐generation sensors and actuators. To this end, kirigami's design and fabrication principles present a significant advantage. Since kirigami starts from a thin, flat material, it is easy to embed electrical and biochemical components at different scales. These nonmechanical components can then deform and reorient with the host kirigami sheet to achieve the desired functions. In addition, the flexibility of host kirigami can ensure their non‐mechanical components (typically stiff) perform robustly even under considerable strain. The following subsections detail some of the well‐investigated electro‐mechanical applications of kirigami.

#### 3.3.1 Optical Tracker and Reflector

**Figure 9:**
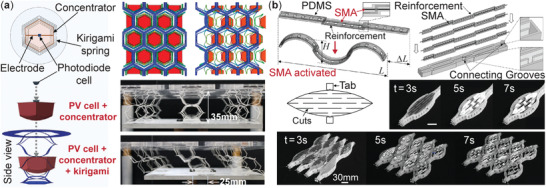
Optical tracking and reflection control with kirigami. a) A multi‐axis solar tracker with embedded photo‐diode cells and concentrator.^[^ ^145^ ^]^ This design can be considered as an evolution from the single‐axis solar tracker shown in Figure 3c. Adapted with permission.^[^ ^145^ ^]^ Copyright 2020, Wiley‐VCH. b) A deployable, SMA‐actuated system with kirigami reflector. Adapted with permission.^[^ ^146^ ^]^ Copyright 2017, Wiley‐VCH.

Traditionally, flat solar panels are coupled with optical tracking systems to maximize electrical power generation over a day. However, these trackers can be complex and require costly and cumbersome structural components.^[^ ^143^ ^]^ To address this challenge, Lamoureux et al. pioneered the use of soft and deforming kirigami as the host structure for solar tracking.^[^ ^18^, ^144^ ^]^ This concept exploits kirigami's programmable kinematics and super‐stretchability properties (reviewed in Sections 2.1 and 2.3). First, thin‐film solar cells are embedded in a host kirigami with parallel cuts. Then, by lifting/lowering the kirigami's free end and stretching it to induce out‐of‐plane deformation, one can control the orientation of the integrated solar cells so they can have the largest projected area from the sun (Figure 3c). Evke et al. later expanded this concept to provide two‐axis solar tracking by using an array of discontinuous and concentric hexagonal cut patterns (**Figure** **9**a).^[^ ^145^ ^]^ These concentric cuts can deform into a spring‐like compliant mechanism when the facets at the center are displaced in the out‐of‐plane z‐direction. By further sliding the center along the x and y‐axis, one can achieve tilting configuration with a controllable angle for the embedded solar concentrators. Such a simple tracking setup can ensure a stable energy output regardless of the changing sun position.

Nonetheless, the solar tracking systems mentioned above still require external mechanical force to achieve their functions. Wang et al. proposed a self‐actuated kirigami reflector by embedding shape memory alloy (SMA) actuators into a PDMS‐based host structure (Figure 9b),^[^ ^146^ ^]^ enabling stretching by simple electric current inputs. Therefore, one can embed solar cells or optical reflectors in this SMA‐enabled kirigami actuator to achieve autonomous solar tracking or optical beam steering. Based on careful cutting pattern design, the kirigami‐based systems can exhibit an extensive range of continuous and controllable tracking angles while being simple, compact, lightweight, and low cost.

#### 3.3.2 Wearable and Wireless Sensors

Our professional athletic industries, human‐robot collaboration, medical rehabilitation, and personal health monitoring efforts demand more capable sensors to accurately measure human subjects' body motions, especially the movement of critical joints like the elbow and shoulder. However, developing wearable sensors for such tasks is challenging because they must accommodate an extensive motion range, adhere to the human body to ensure accuracy, and avoid hindrance to the wearers' everyday activities.^[^ ^147^, ^148^ ^]^ Traditionally, motion tracking is achieved based on camera tracking systems^[^ ^149^ ^]^ and inertial measurement units (IMUs)—consisting of an accelerometer, gyroscope, and magnetometer—packaged in a rigid brace or suit. To this end, kirigami can be an ideal host structure for constructing wearable and flexible sensors due to its super‐stretchability and programmable kinematics.

**Figure 10:**
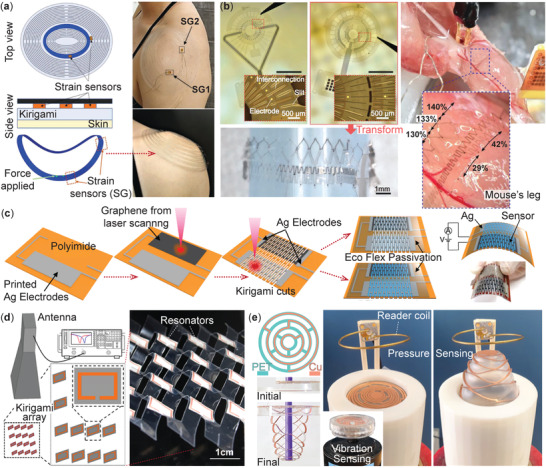
Flexible and wireless sensors based on the kirigami principle. a) A wearable kirigami patch with embedded strain gauges can measure the wearer's shoulder joint movements. Adapted with permission.^[^ ^150^ ^]^ Copyright 2019, Wiley‐VCH. b) A donut‐shaped, kirigami‐based bio‐probe can track mouse muscle's EMG signals. Adapted with permission.^[^ ^71^ ^]^ Copyright 2019, Wiley‐VCH. c) An integrative fabrication procedure for making flexible sensors with kirigami cut graphene and Ag electrodes. Notice that the passivation step can significantly improve the sensor's durability. Adapted with permission.^[^ ^68^ ^]^ Copyright 2019, Royal Society of Chemistry. d) A wireless strain sensor with embedded split‐ring resonators on the kirigami facets. Adapted with permission.^[^ ^85^ ^]^ Copyright 2020, American Chemical Society. e) A mechanically stable kirigami sensor with embedded copper coils for different tasks, such as vibration and pressure sensing.Adapted with permission.^[^ ^151^ ^]^ Copyright 2021, American Chemical Society.

There are two different approaches to using kirigami for wearable sensors. The first approach is placing traditional sensors on a host kirigami and adhering them to the wearer's body by exploiting the super‐stretchability.^[^ ^76^, ^152^ ^]^ For example, Erin et al. turned a rotationally symmetric kirigami with embedded strain sensors into a conformal patch, which can provide real‐time tracking of shoulder joint movements (**Figure** **10**a).^[^ ^150^ ^]^ Morikawa et al. developed a donut‐shaped kirigami bio‐probe device to measure the electromyography (EMG) signal of muscle tissues.^[^ ^71^ ^]^ The super‐stretchability of the host kirigami ensures the probe's robust performance regardless of the muscle deformation (Figure 10b). It is also worth noting that the nonsmooth surface of stretched kirigami can improve the adhesion between the sensor and biological tissue, ensuring accurate readings.^[^ ^153^ ^]^ Yong et al. designed a wearable kirigami‐inspired graphene electrode that exhibits strain‐insensitive electrical properties up to 240% of tensile strain with different strain states, including stretching, twisting, and shearing.^[^ ^154^ ^]^ The out‐of‐plane buckling near kirigami cuts strategically re‐distributed stress concentrations away from the active sensing elements.

Another approach is directly applying kirigami cuts onto the sensors (or sensing materials) to make them flexible.^[^ ^155^ ^]^ For example, Xu et al. developed a laser‐treated, graphene‐based, stretchable strain sensor on a polyimide kirigami film (Figure 10c).^[^ ^68^ ^]^ Here, the kirigami cuts give graphene enough flexibility so that it can change its electric resistance in response to stretching deformation. This strain sensor (including the polyimide base layer, responsive graphene layer, and Ag electrodes at the ends) is passivated with Ecoflex polymer to ensure consistent sensing capability even after more than 60 000 loading cycles.

Besides the wearable sensors, we are also witnessing an increasing interest in other kirigami‐based sensors, especially wireless sensors for noninvasive strain measurement in structural health monitoring and bio‐medical applications.^[^ ^156^, ^157^, ^158^ ^]^ For example, Dijvejin et al. designed a highly sensitive wireless strain sensor by embedding an array of split‐ring resonators (SRRs) in a parallel‐cut kirigami host structure (Figure 10d).^[^ ^85^ ^]^ The idea is that, as the kirigami deforms, the resonators will rotate with the facets and change their resonance frequency with respect to a fixed incoming microwave. A transceiver horn antenna can measure such resonance frequency shifts as it continuously sends microwave signals with swept frequencies and measures the reflected energy. Resonance occurs when the reflected energy is minimum. The kirigami‐based wireless sensor is precise, up to 10% of strain. Similar wireless strain sensor concepts have been developed with different levels of success.^[^ ^159^, ^160^ ^]^

Another unique wireless sensor design integrates thin metallic strips into a polymer film with a hinge‐connected concentric circle cut pattern (Figure 10e).^[^ ^151^ ^]^ When this kirigami is deformed out‐of‐plane, generating a helical shape, the embedded metallic strip becomes an LCR circuit that can perform as a wireless sensor for different inputs like displacement, acceleration, or pressure. For example, one can embed a seismic mass at the sensor's center and place it inside a reader coil. This coil generates an electromagnetic field with a sweeping frequency via a controlled input current. By exploiting the near‐field inductive coupling between the reader coil and metallic strips in kirigami, one can accurately measure acceleration by reading the peak‐peak amplitude inside the sensor. In summary, due to the vast freedom of programming kirigami's mechanical responses and embedding different electrical components, there are many untapped potentials in wearable and wireless kirigami sensors.

It is also worth noting that besides sensors, there is also a rising interest in infusing similar kirigami principles into energy harvesters, especially those based on piezoelectric materials, to improve their performance.^[^ ^32^, ^74^ ^]^

### 3.4 Robotics

We also witness an increasing interest in using kirigami as functional components for robotic systems, especially soft robots. Soft robots, as their name implies, are constructed based on compliant materials. Their body's softness makes them versatile in dynamically constrained working conditions, inherently safe for human–robot interactions, and potentially less expensive to fabricate and transport than rigid‐bodied robots.^[^ ^161^, ^162^ ^]^ Therefore, soft robots have been considered the next significant evolution in the robotics discipline. Kirigami is naturally an ideal candidate for soft robotic skeletons due to its programmable kinematics, tunable mechanical properties, and ease of integrating electronic components, micro/nanoscale sensors, and actuators.^[^ ^99^, ^163^, ^164^ ^]^

**Figure 11:**
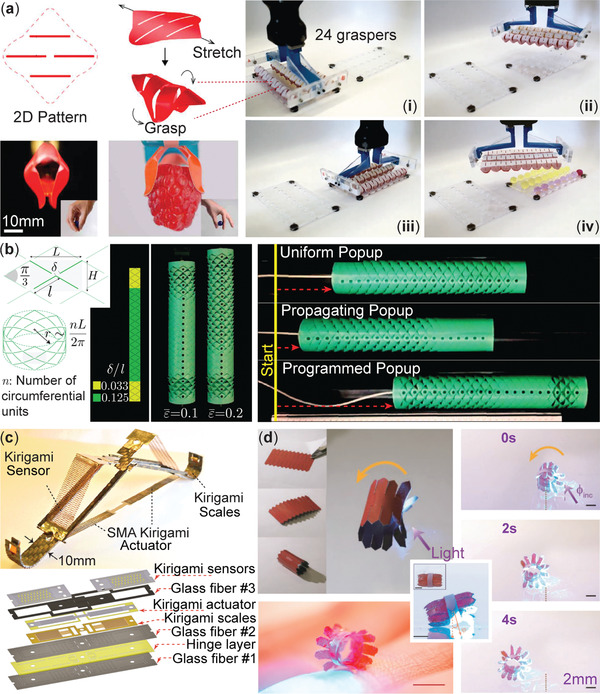
Kirigami‐based robots. a) A scalable gripper based on carefully designed kirigami shells that can transform a mechanical stretching input into a grasping motion. Such a design is ideal for manipulating soft objects like hydrogel balls shown on the right. Adapted with permission.^[^ ^19^ ^]^ Copyright 2021, AAAS. b) A kirigami‐skinned crawling robot. The cut pattern in this skin is nonuniform, so one can program the pop‐up (or buckling) sequence of its triangular facets, allowing the robot to locomote faster. Adapted with permission.^[^ ^165^ ^]^ Copyright 2019, National Academy of Science. c) An SMA‐based robot with integrated actuators, sensors, and scales, all leveraging kirigami design principles. Adapted with permission.^[^ ^99^ ^]^ Copyright 2020, IEEE. d) A robot that can deform and row in response to input light. Adapted with permission.^[^ ^131^ ^]^ Copyright 2019, Wiley‐VCH.

The two most fundamental tasks for soft robots are object manipulation and locomotion. Regarding the former task, soft robotic manipulators have exceptional adaptability, allowing them to grasp and handle objects with irregular shapes autonomously.^[^ ^166^, ^167^ ^]^ By exploiting the advantages of kirigami, engineers have designed simple yet robust soft grippers capable of precise and rapid grasping. For example, Yang et al. developed a shell‐like kirigami gripper actuated by simple stretching (**Figure** **11**a).^[^ ^19^ ^]^ Its parallel cut pattern is designed carefully to direct its out‐of‐plane bending for effective gripping motion, allowing it to pick up a wide range of objects, including raspberry, wallet, and tiny sand grains. In a different study by Tang et al., a self‐folding kirigami gripper was made by bonding three layers of materials (paper‐shape memory polymer‐paper) with patterned cuts. The middle layer of SMP can expand or shrink in response to external thermal stimuli.^[^ ^29^ ^]^ In this way, we can use the actively controllable kirigami gripper to capture objects in places inaccessible to humans or traditional machines.

Kirigami also opens avenues to a new class of soft robots that can locomote in complex environments for rescue, exploration, environmental monitoring, and biomedical tasks. For example, Rafsanjani et al. designed a kirigami‐based flexible skin that can be integrated with a fiber‐reinforced pneumatic body to achieve snake‐like crawling locomotion.^[^ ^140^ ^]^ While other soft robots typically require multiple actuators to achieve locomotion,^[^ ^168^, ^169^ ^]^ this kirigami‐based robot only needs one actuation input: By simply inflating the body, the kirigami skin will be stretched and exhibit buckling (Section 2.4). As a result, the scale‐like kirigami facets “pop up” outward to create directional (asymmetric) friction and move the robot forward (Figure 11b). The kirigami skin design was later evolved with a nonuniform heterogeneous cut pattern.^[^ ^165^ ^]^ Such intentional nonuniformity can program the pop‐up sequence among different parts of kirigami skin—much like sequencing a locomotion gait—to double the locomotion speed. Inspired by the locomotion capabilities of snakes, Branyan et al. further introduced scoring along the longitudinal axis in a polyester kirigami skin. They experimentally demonstrated that such skin could provide lateral resistance to achieve undulation locomotion across smooth and textured surfaces.^[^ ^170^ ^]^ It is worth noting that the directional friction from the stretch‐buckled and popped‐up kirigami has seen other applications. For example, Liu et al. fabricated a kirigami‐skinned earthworm robot with a more robust anchoring with loose soil from the bristle‐like facets.^[^ ^139^ ^]^ Babaee et al. attached steel‐based kirigami patches to the shoe soles to reduce the risk of slips and falls in different environments.^[^ ^64^ ^]^

Besides crawling, legged locomotion is also possible with kirigami robots. For example, based on reconfigurable kirigami with multiple degrees of freedom, Tang et al. demonstrated a legged kirigami robot capable of walking and turning.^[^ ^29^ ^]^ Firouzeh et al. designed a more integrated, mesoscale robot by embedding sensing, actuation, and control in its kirigami body (Figure 11c).^[^ ^99^ ^]^ It has three kirigami components, each playing a unique role according to its electrical, thermal, and mechanical properties: a stretchable sensor to measure body motion, a shape memory alloy (SMA) kirigami to provide actuation, and pop‐up kirigami scales to control locomotion direction. The multiple design parameters in kirigami provide a vast design space with the potential to meet requirements from different robotic applications.

Besides mechanical stretching and embedded shape memory alloys (or polymers), there are a few other remote actuation strategies for kirigami robots involving responsive materials that can respond to light,^[^ ^131^ ^]^ heat,^[^ ^130^ ^]^ and chemical solvent.^[^ ^171^ ^]^ For example, via attaching light‐responsive liquid crystal polymer (LCN) to the host kirigami, Cheng et al. designed a rolling robot activated by a light beam (Figure 11d).^[^ ^131^ ^]^ By controlling the light beam's irradiation intensities and direction, the robot can change its locomotion modes, turning, climbing, and navigating on pre‐determined routes. The number of papers on kirigami robotics is much smaller than on other topics like super‐stretchability; therefore, we believe robotics will be another emerging topic in the engineering applications of kirigami principles.

## 4 Outlook and Future Challenges

This review details the recent progress of kirigami‐inspired engineering systems regarding their geometric design, mechanical properties, underlying mechanics, and rich functionalities. Undoubtedly, the ancient art of paper cutting will continue to inspire innovative engineering research in the future. In particular, the kirigami principle is widely applicable to mechanical metamaterials, flexible electronics, and soft robotics, all of which are emerging disciplines with significant impacts on our strategic industries and daily lives. Therefore, there is tremendous potential for exploiting the cutting‐induced mechanical properties and multi‐disciplinary functionalities reviewed in this paper. Nonetheless, a few challenges and open topics remain to be addressed.

First, studies on dynamic functionalities in kirigami are still an open field, especially regarding wave propagation control. So far, we have only seen a few proof‐of‐concept studies on exploiting the periodic geometries of kirigami for flexural wave control (either waveguide or stopband). However, the versatility of kirigami can open up exciting new opportunities. For example, one could leverage the topological mechanics of kirigami to create topologically protected waves,^[^ ^119^, ^172^ ^]^ carefully design nonuniform cut patterns to develop metasurfaces,^[^ ^173^ ^]^ harness geometric nonlinearity to achieve non‐reciprocal wave propagation,^[^ ^174^ ^]^ and embed simple electronics to accomplish mechanical computation.^[^ ^175^, ^176^ ^]^ The vibrant research activities on relevant topics—throughout engineering, condensed matter physics, and computing science communities—can serve as the inspiration and guidelines for kirigami‐based studies.

Another open topic worth investigating is an inverse design methodology that uses different mechanical properties and multi‐functionality as the target. Remarkably, most of the kirigami patterns reviewed in this paper are derivatives of the two archetypal cut patterns—parallel and cross cuts—shown in Figure 1. These underlying cutting pattern designs are clever but also ad hoc. On the other hand, the currently available design methods reviewed in Section 2.1 can only handle kinematics (aka shape morphing). The possibilities of distributing cuts in a kirigami sheet are endless, so there is a crucial need for an inverse design method to generate kirigami cuts to achieve prescribed functions beyond shape morphing. To formulate such a design method, one might adapt and evolve other well‐established design tools such as topology optimization^[^ ^30^, ^177^ ^]^ or machine‐learning‐based methods.^[^ ^178^, ^179^ ^]^

Finally, the long‐term success of kirigami‐inspired engineering relies upon the in‐depth synergy between different branches of research and “real‐world” implementations. The current state of the art focuses on revealing the potential of kirigami‐inspired systems and their applications. Developing and maturing real‐world applications in which kirigami can outperform other technologies is crucial for this research topic's long‐term sustainability. It requires a deep integration of our knowledge in design, mechanics modeling, scalable fabrication, and material science.

## Conflict of Interest

The authors declare no conflict of interest.
